# Stereoacuity with Frisby and Revised FD2 Stereo Tests

**DOI:** 10.1371/journal.pone.0082999

**Published:** 2013-12-12

**Authors:** Iwo Bohr, Jenny C. A. Read

**Affiliations:** TThe Institute of Neuroscience, Henry Welcome Building for Neuroecology, Newcastle University, Newcastle upon Tyne, United Kingdom; University of Muenster, Germany

## Abstract

We compared near stereoacuity, measured with the Frisby test, and distance stereoacuity, measured with the revised Frisby-Davis (FD2) test, enabling a comparison with the original version of the FD2. In the revised version of the FD2 test, a white background is used instead of a backlit background. We also examined the effect of age, gender and visual problems. We used the Frisby test at distances ranging from 30–80 cm and FD2 at 6 m. The best possible score was 20 seconds of arc (arcsec) on the Frisby and 5 arcsec on the FD2; participants who could not perform a test despite demonstrating understanding of it were classed as stereonegative. We examined both the whole population recruited, and a sub-population screened so as to exclude visual problems. We analysed our results in three age-groups: “visually developing” (36 children aged 5–10 years); “visually mature” (300 participants aged 11–49 years) and “older” (29 participants aged 50–82). In the whole population, the median stereoacuity on the Frisby test was 25, 20 and 85 arcsec in the three age-groups. In the sub-population with no visual problems, median Frisby stereoacuity was similar at 20, 20 and 80 arcsec respectively. On the FD2, the medians were 10, 10, 20 arcsec for the whole population and 7.5, 10 and 12.5 for the sub-population. Children were more likely than adults to be stereonegative on the FD2, although none of the children were stereonegative on the Frisby. The two tests showed fair agreement when used to classify people into three categories of stereovision. Poor stereovision was often associated with binocular problems such as tropia, but with many exceptions. In line with previous studies, we found improvements in measured stereoacuity in childhood and declines in late adulthood. The new FD2 test gives comparable values to the original FD2.

## Introduction

Binocular stereopsis refers to the perception of depth from binocular disparity. Stereopsis emerges early on in development at 3 to 6 months of life [1]–[8], continues to mature until about 10 years of age [1], [9]–[11] and declines in later life [12]–[17].

Stereopsis is typically assessed by measuring stereoacuity: the smallest “threshold” disparity which can be discriminated between two adjacent surfaces. Stereoacuity depends both on cortical mechanisms sensitive to retinal disparity, and also on the control of eye movements, since optimal stereoacuity is achieved when both eyes fixate reliably on the location of the disparity step between the surfaces. Stereoacuity is an important clinical tool in screening for strabismus or amblyopia, though it may not always be effective [18]. Measured stereoacuity may be used as an indicator for intervention to correct strabismus, including surgery [19]–[22], and as an outcome measure in assessing the effectiveness of treatment [22], [23].

Four stereo tests which have been developed for clinical use are the TNO, Randot, Frisby and Frisby-Davis Distance (FD2) tests. The FD2 test is unusual in that it is designed to test stereoacuity with distance viewing (>3 m). Distance stereoacuity is particularly important clinically, because clinical groups such as intermittent exotropes are more impaired on distance than near stereoacuity [24], [25]. The FD2 test has recently become available in a modified form, omitting the backlight contained in the original version. Test norms for this new FD2 test are not yet available. Furthermore, published results for the Frisby and original FD2 tests often involve relatively small numbers of subjects, e.g. 20 [26], 36 [27], 59 [28], 22 [29], 92 [30], 73 [21], 95 [31], 140 [32]. Ohlsson and co-workers[18] screened an impressive 1035 children, but only report stereoacuity for 60 children with strabismus and/or amblyopia.

As part of a wider study, we have recently acquired a large data-set on near stereoacuity measured by the Frisby test and on distance stereoacuity measured by the FD2. The 365 participants were recruited from the general public and tested by qualified orthoptists. We were interested in examining stereoacuity in daily life in the general population, not in a “normal” population defined by the absence of clinical pathology. Accordingly, we measured stereoacuity using participants’ habitual correction, and did not exclude participants based on visual pathology, although we do report stereoacuity separately for participants with visual problems. In contrast to other studies, this work is characterized by a large cohort in the 11–49 age-ranges, a wide total age-range (5–82 years old) and the fact that scores on two stereo tests are available. Additionally, this data-set represents the first published results for the revised FD2 test, and thus the first comparison between the revised FD2 and the Frisby stereo tests.

## Methods

### Ethics Statement

The study was approved by the Newcastle University Faculty of Medical Sciences Ethics Committee and adhered to the tenets of the Declaration of Helsinki. All participants, or in case of children, adults with parental responsibility, gave written informed consent.

### Participants

**Figure 1:**
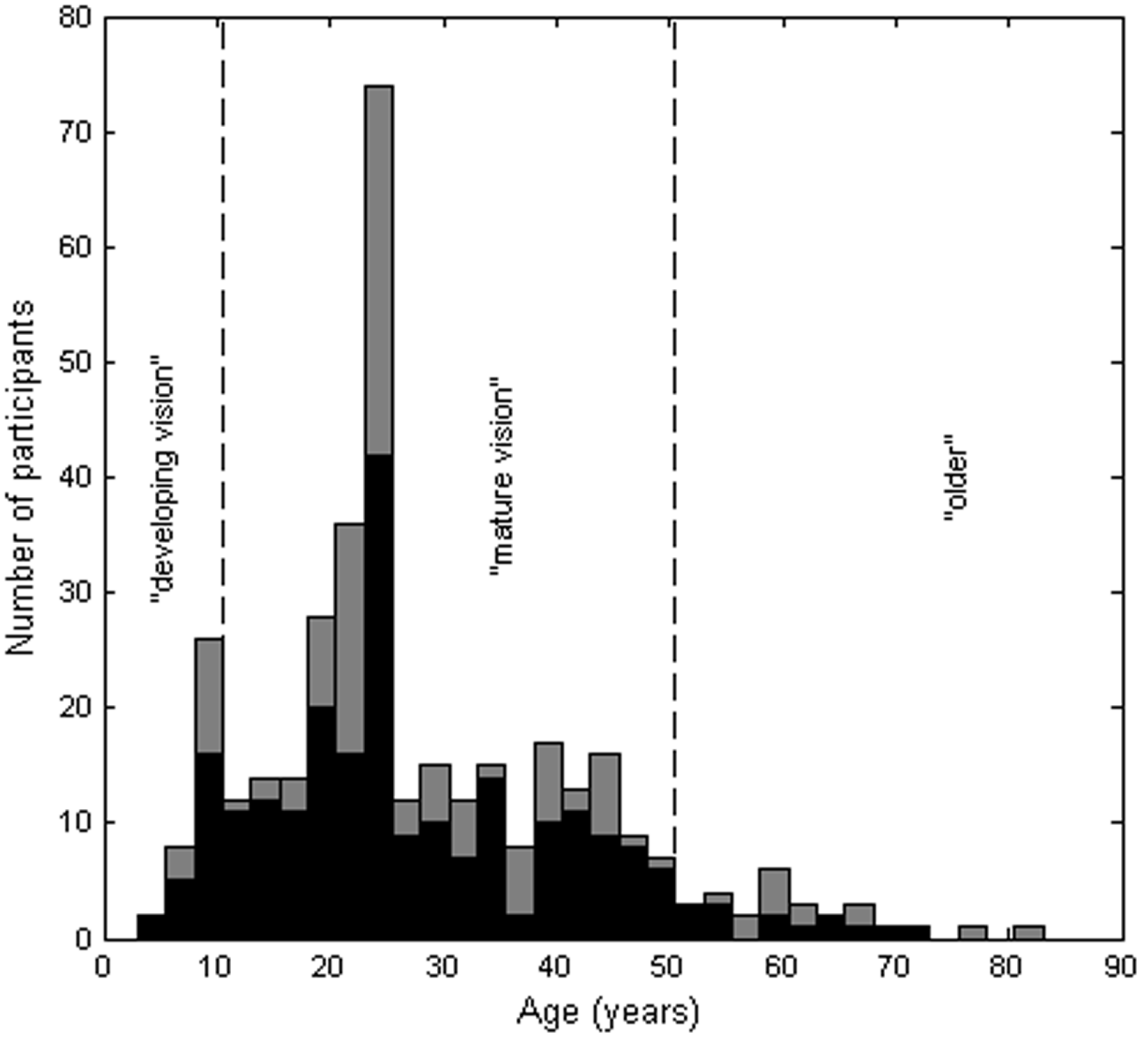
Frequency histogram of participant ages, in bins of width 2.5 years. Vertical dashed lines separate the three age-subgroups examined. Dark bars indicate participants with no visual problems; lighter bars show participants who may have had a visual problem (see Methods for details). Note that in order to preserve anonymity, only year of birth was recorded. For brevity, “age” in this paper means “year of birth subtracted from year of testing”.

The distribution of participant ages is shown in Figure 1. Our sampling was not representative of the UK population, with a clear bias towards participants in their early twenties. Not all participants completed both tests and gender information was not recorded for 12 participants (for detailed information, see Tables 1 and 2). Participants wore their usual visual correction for the stereo tests.

**Table 1:** Stereo thresholds on the Frisby test for different groups.

Near stereoacuity with Frisby stereo test	All ages	“visually developing” (5–10)		“visually mature” (11–49)			“older” (50–82)
Whole population sampled							
N tested	365		36		300	29	
Age in years, mean ± SD	27.9±14.3		8.4±1.4		27.1±9.7	60.4±7.9	
N stereonegative (%)	5 (1%)		0 (0%)		3 (1%)	2 (7%)	
**Median stereo threshold (inter-quartile range)**	**20 (20**–**40)**		**25 (20**–**40)**		**20 (20**–**40)**	**85 (20**–**120)**	
Median stereo threshold for males	20 (N = 140)		30 (N = 18)		20 (N = 106)	42.5 (N = 16)	
Median stereo threshold for females	20 (N = 213)		20 (N = 17)		20 (N = 183)	85 (N = 13)	
Sub-population with no visual problems							
N tested	235		23		196	16	
Age in years, mean ± SD	27.4±13.9		8.3±1.6		27.1±10.3	58.3±7.2	
N stereonegative (%)	0 (0%)		0 (0%)		0 (0%)	0 (0%)	
**Median stereo threshold (inter-quartile range)**	**20 (20**–**30)**		**20 (20**–**40)**		**20 (20**–**30)**	**80 (20**–**130)**	
Median stereo threshold for males	20 (N = 94)		20 (N = 9)		20 (N = 76)	75 (N = 9)	
Median stereo threshold for females	20 (N = 133)		20 (N = 13)		20 (N = 113)	85 (N = 13)	
Sub-population with potential visual problem							
N tested	130		13		104	13	
population wAge in years, mean ± SD	28.9±15.0		8.5±1.1		27.2±8.5	63.1±8.1	
N stereonegative (%)	5 (4%)		0 (0%)		3 (3%)	2 (15%)	
**Median stereo threshold (inter-quartile range)**	**30 (20**–**55)**		**30 (20**–**51.2)**		**20 (20**–**40)**	**85 (30**–**120)**	
Median stereo threshold for males	30 (N = 46)		40 (N = 9)		30 (N = 30)	30 (N = 7)	
Median stereo threshold for females	20 (N = 80)		20 (N = 4)		20 (N = 70)	97.5 (N = 6)	

“N tested” refers to the number of participants who were examined and who demonstrated an ability to understand the test and cooperate with the tester. “N stereonegative” refers to the number who could not perform the test at the largest disparity available, despite a demonstrated ability to understand the test and cooperate. Note: the number of males and females does not sum to the total N in the group, since gender was not recorded for all participants. All ages are in years and all stereo thresholds are in seconds of arc. Medians are followed by the interquartile range in parentheses.

**Table 2:** Stereo thresholds on the FD2 test (in seconds of arc).

Distance stereoacuity with FD2 stereo test	All ages		“visually developing” (5–10)			“visually mature” (11–49)		“older” (50–82)
Whole population sampled								
N tested		363		35	299		29	
Age in years, mean ± SD		28.0±14.3		8.5±1.4	27.1±9.7		60.4±7.9	
N stereonegative (%)		10 (3%)		4 (11%)	4 (1%)		2 (7%)	
**Median stereo threshold (inter-quartile range)**		**10 (5**–**20)**		**10 (5**–**18.8)**	**10 (5**–**20)**		**20 (10**–**31.2)**	
Median stereo threshold for males		10 (N = 139)		10 (N = 17)	10 (N = 106)		22.5 (N = 16)	
Median stereo threshold for females		10 (N = 212)		10 (N = 17)	10 (N = 182)		20 (N = 13)	
Sub-population with no visual problems								
N tested		233		22	195		16	
Age in years, mean ± SD		27.5±13.9		8.5±1.6	27.1±10.3		58.3±7.2	
N stereonegative (%)		1 (0.4%)		1 (5%)	0 (0%)		0 (0%)	
**Median stereo threshold (inter-quartile range)**		**10 (5**–**15)**		**7.5 (5**–**15)**	**10 (5**–**15)**		**12.5 (10**–**27.5)**	
Median stereo threshold for males		10 (N = 93)		7.5 (N = 8)	10 (N = 76)		15 (N = 9)	
Median stereo threshold for females		10 (N = 132)		5 (N = 13)	10 (N = 112)		20 (N = 13)	
Sub-population with potential visual problem								
N tested		130		13	104		13	
Age in years, mean ± SD		28.9±15.0		8.5±1.1	27.2±8.5		63.1±8.1	
N stereonegative (%)		9 (7%)		3 (23%)	4 (4%)		2 (15%)	
**Median stereo threshold (inter-quartile range)**		**15 (10**–**25)**		**15 (13.8**–**172.5)**	**15 (7.5**–**25)**		**25 (17.5**–**45)**	
Median stereo threshold for males		20 (N = 46)		15 (N = 9)	20 (N = 30)		25 (N = 7)	
Median stereo threshold for females		15 (N = 80)		stereo-negative (N = 4)	15 (N = 71)		32.5 (N = 6)	

Table 1. Details as for

We analyzed the whole data-set, and also compared stereovision in three age-subgroups, marked with vertical lines in Figure 1:

- 5–10 years old (“visually developing”, n = 36):
- older than 10 years and younger than 50 (“visually mature”, n = 300)
- aged 50 or over (“older”, n = 29)

This subdivision into age-groups enabled us to compare participants who had reached visual adulthood with those whose visual systems were still developing and those who might be affected by the decline in stereoacuity due to aging [13]–[17], [33], [34]. 4 additional children under 5 in our original data set were excluded from the main analysis due to the small sample size in this age-group. We did not further subdivide the “visually mature” group for purposes of presentation here, since our data showed no change in stereo thresholds over this age-range (see Results).

### Visual acuity

Participants were asked to undergo an optometric and orthoptic examination. These took place at C4 Sightcare’s optometry practices in Newcastle upon Tyne or Morpeth (www.C4sightcare.com). Visual acuity data is especially relevant for this study, since stereovision may be impaired either by low acuity, or by a large interocular acuity difference. For participants aged 8 and over, refractive error was first measured by the optometrist at 0.4 m and 6 m, and then visual acuity was measured with the best optical correction at both distances. Visual acuity was also measured at 6 m with participants wearing their habitual correction. At 0.4 m, visual acuity was measured using the printed Sussex LogMAR test (Sussex Vision International, UK; http://www.sussexvision.co.uk); at 6 m, it was measured using the Thomson LogMAR test on a computer (Thomson Software Solutions, UK; http://www.thomson-software-solutions.com). In every case, acuity was measured with right and left eyes monocularly using an occluder and then binocularly. This resulted in a total of 9 acuity measurements for participants aged 8 and over.

For the 0.4 m Sussex LogMAR test the room was illuminated during the test and participants were asked to identify the individual letters on each line starting from the largest. Series Charts 1 and 2 were presented to the right and left eyes respectively and Series Chart 1 was used binocularly. Individual letters correctly identified were included in the recording sheet and used to determine the logMAR acuity. For the 6 m Thomson LogMAR test the room was illuminated during the test and participants were asked to identify the individual letters on each line starting from the largest. A randomised letter sequence was displayed on a computer monitor. Individual letters correctly identified were entered on the recording sheets and used to determine the logMAR acuities.

For participants under 8 years old, visual acuity was measured by the orthoptist at 3 m using the Keeler logMAR test (Keeler Ltd; http://www.keeler.co.uk), wearing habitual correction. The room was illuminated during the test. The screening test was used to determine which line to begin with. Children who knew their letters read out the letters across each line without prompting. A matching card could be used where letters were not known, and the orthoptist could point to each letter in turn with a pointer, taking care to ensure they did not encroach on the crowding box. Testing was continued until the participant could not identify all the letters on the line correctly. The acuity was then recorded based on the number of letters correctly identified. To test monocularly, occluder glasses were used, or for participants who already wore glasses, patches could be taped over their glasses. A different book was used for each eye. This resulted in 3 separate visual acuity measures from participants aged under 8. Due to limited cooperation, it was not always possible to make all measurements in child participants.

### Stereoacuity tests: general information

Stereovision tests were carried out by qualified orthoptists. The measured outcome was the lowest disparity a participant could reliably distinguish. Two out of three correct choices were required on the smallest disparity. In the event of uncertainty, the test was repeated. Both the Frisby and FD2 tests use real depth, and thus allow for non-stereo cues. It is therefore possible in principle to pass these tests monocularly [35]. To avoid this, participants were retested monocularly. If they obtained the same score monocularly as binocularly, they were classed as stereonegative. Previous work indicates that this protocol avoids monocular participants passing the test [31].

### Frisby Stereo Test

This test is used to assess stereovision at closer distances, requiring eye convergence. For a detailed description see Simons [30]; and Frisby et al. [36] Briefly, the participant’s task is to detect a circle containing a pattern of geometric objects (the target) visible within a mosaic of similar geometric shapes. The target and background are printed on opposite sides of a Perspex plate, and so differ in their physical depth. The angular disparity depends on the thickness of the plate and the distance from the observer. The Frisby test comprises three plates, each of which can be presented at one of several different possible distances to obtain a range of disparities. Our protocol did not specify whether the plates were presented with crossed or uncrossed disparity. Thresholds have been reported to be the same for both [13]. Our orthoptists used test distances ranging from 30 to 80 cm, yielding the disparities shown in Table 3. The available disparities ranged from 20 arcsec (well above the best achievable threshold for normal subjects) to 600 arcsec. Participants who could not identify the target at 600 arcsec were classed as stereonegative. Adams et al. [27] found that the 95% limit for test-retest agreement was 0.24 log arcsec (a factor of 1.74) on the near Frisby test, making it the most reliable of the 4 stereo tests they examined. To avoid the use of monocular cues, the orthoptist ensured that the participant had the plates directly in front of them and kept their head still.

**Table 3:** Viewing distances and resulting disparities (arcsec) for the Frisby stereo test, reproduced from the test documentation.

	Plate thickness		
Viewing distance	6 mm	3 mm	1.5 mm
30 cm	600	300	150
40 cm	340	170	85
50 cm	215	110	55
60 cm	150	75	40
70 cm	110	55	30
80 cm	85	40	20

### Frisby-Davis 2 Test for stereoacuity at a long distance (FD2)

This test is described by Adams et al [28]. Like the Frisby test, it uses physical depth. Briefly, the participant views four shapes (star, cross, arrow, crescent) attached to a box at a distance of 6 m. The shapes are mounted on horizontal rods which enable them to be slid towards or away from the viewer. With the door of the box closed, so the subject cannot see the movement, one shape is moved nearer to the viewer. The door is then opened and the subject is asked which shape is nearer. In this test, the possible disparities ranged from 5 to 50 arcsec in steps of 5 arcsec. Lower thresholds could thus be obtained on the FD2 compared to the Frisby test. Participants who could not discriminate the largest disparity despite demonstrating understanding of the test were classed as stereonegative. Adams et al. [27] found that the 95% limit for test-retest agreement was 0.68 log arcsec (a factor of 4.8) on the original FD2 test, making it the least reliable of the 4 stereo tests they examined. Since the modified version of the test relies on natural light (absence of backlight), the test was positioned so as to prevent shadows which could be used as monocular cues.

### Classifying stereoacuity

Comparing the two tests is complicated by the different range of scores possible on each test in our study, notably the large minimum threshold (20 arcsec) on the Frisby test imposed by our use of a maximum viewing distance of 80 cm, and the low maximum threshold (50 arcsec, or stereonegative) on the FD2. The two tests probe different aspects of stereopsis; they do not provide alternative means of measuring a common stereo threshold, and so do not agree on a Bland-Altman analysis. However, it is still useful to ask whether they agree more qualitatively. In the clinic, the tests are often used to classify a particular patient as having normal or impaired stereopsis, rather than necessarily to obtain a precise threshold, so it is important to understand whether the tests agree on this qualitative judgment. To assess this, we simplified each data-set by dividing stereovision into 3 classes. We defined Class 1 as a stereo threshold of ≤20 arcsec; this was the best possible score on the Frisby test, and also the median score (Table 1). We defined Class 2 as stereo thresholds above 20 and below 150 arcsec. We defined Class 3 as 150 arcsec and above. Since the FD2 test offers a maximum disparity of 50 arcsec at our test distance of 6 m, all subjects in Class 3 on the FD2 will have tested stereonegative. On the Frisby, subjects in Class 3 might have tested stereonegative, or might have passed with a threshold of 170 arcsec or greater. Tables 4, 5 and 6 show the number of participants in each classification.

**Table 4:** Contingency table showing relationship between visual problems and stereo classification on the Frisby test.

	Number of participants with:		
	no visual problem	possible visual problem	Totals
Frisby Class 1 (θ ≤ 20 arcsec)	156 (66%)	64 (49%)	220
Frisby Class 2 (20 < θ < 150 arcsec)	71 (30%)	55 (42%)	126
Frisby Class 3 (θ ≥ 150 arcsec)	8 (3%)	11 (8%)	19
Totals	235	130	?^2^ significance = 0.003

The significance is under a chi-squared test of association.

**Table 5:** Contingency table showing relationship between visual problems and stereo classification on the FD2 test.

	Number of participants:		
	no visual problem	possible visual problem	Totals
FD2 Class 1: θ ≤ 20 arcsec	203 (86%)	90 (69%)	293
FD2 Class 2: 20 < θ < 150 arcsec	29 (12%)	31 (24%)	62
FD2 Class 3: θ ≥ 150 arcsec	1 (0%)	9 (7%)	10
Totals	234	131	?^2^ significance = 0.00003

Table 4. Details as for

**Table 6:** Number of participants in the 3 classes of stereoacuity, as assessed on the Frisby (rows) and FD2 tests (columns) for 363 participants for whom both tests were available.

Number of participants total N = 363		FD2 Class 1: θ ≤ 20 arcsec	FD2 Class 2: 20 < θ < 150 arcsec	FD2 Class 3: θ ≥ 150 arcsec
Frisby Class 1: θ ≤ 20 arcsec		195 (54%)	22 (6%)	2 (0.6%)
Frisby Class 2: 20 < θ < 150 arcsec		86 (24%)	37 (10%)	2 (0.6%)
Frisby Class 3: θ ≥ 150 arcsec		12 (3%)	1 (0.3%)	6 (2%)

### Definition of visual problems

**Figure 2:**
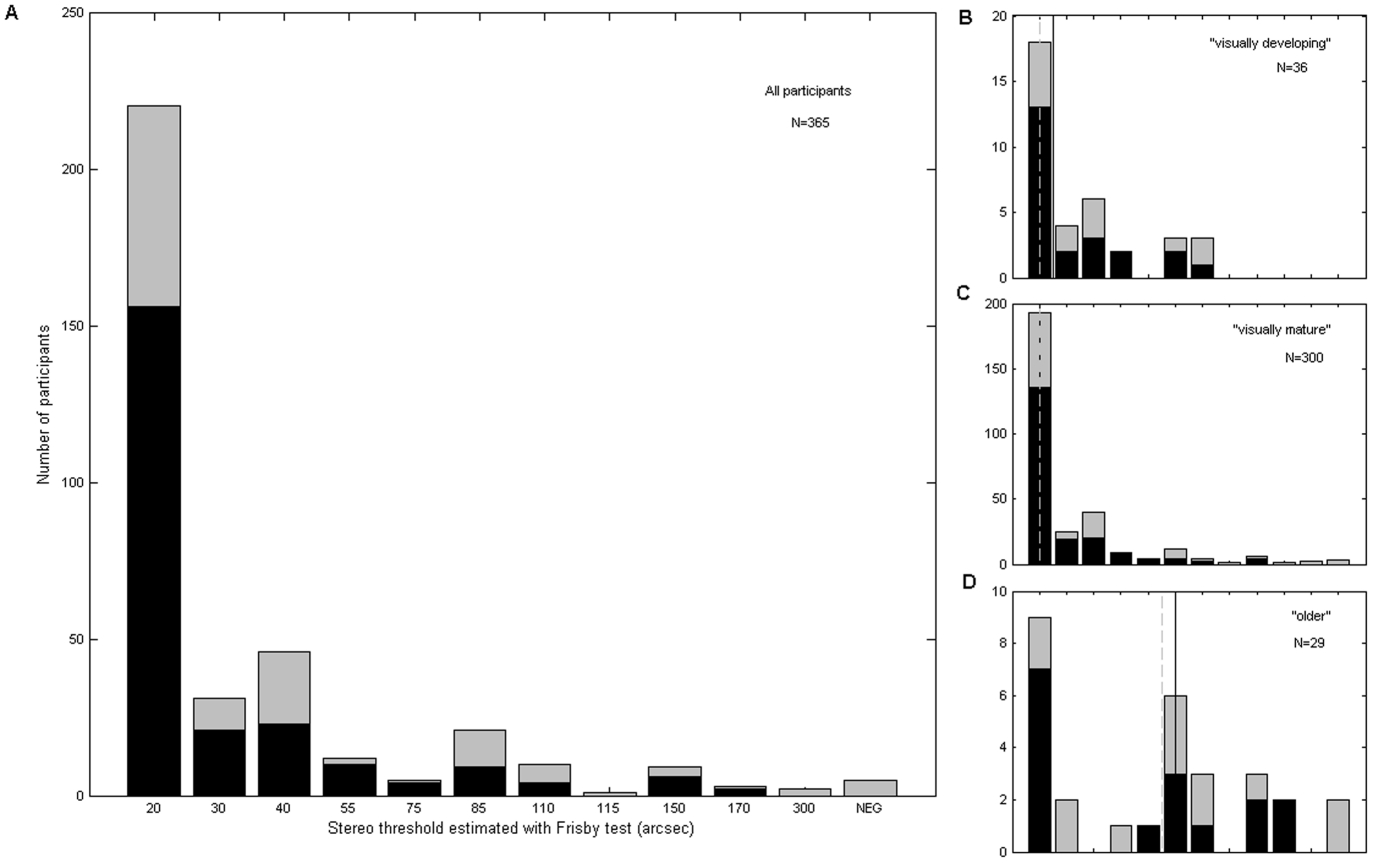
Frequency histogram of stereo thresholds estimated with the Frisby test. NEG  =  stereonegative; unable to perform test at largest available disparity. Main plot: all participants (dark bars  =  “no visual problem”; light bars  =  “visual problem”, as in Figure 1). Subplots: age subgroups on the same horizontal axis. Solid vertical line shows median for “no visual problem” subpopulation (dark bars); dashed vertical line shows median for whole group.

**Figure 3:**
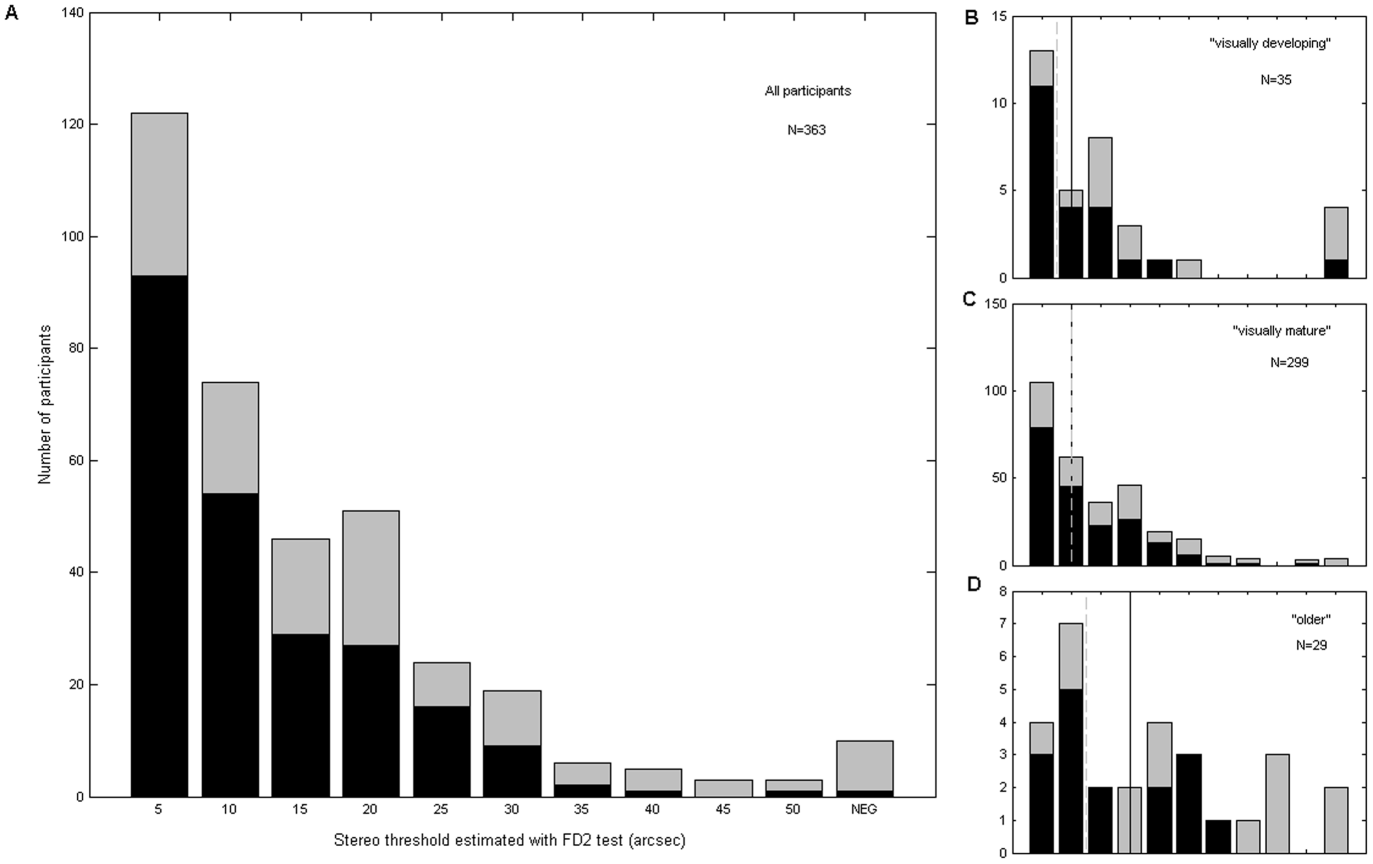
Frequency histogram of stereo thresholds estimated with the FD2 test. Other details as for Figure 2.

In addition to the stereo test data, we also had the results of an optometric and orthoptic examination. This enabled us to analyse participants with particular visual problems separately. In this paper, we report results for the whole population recruited (total bars in Figs 1–3), and also for the sub-population classed as having “no visual problem” (dark bars). Participants were regarded as potentially having a visual problem if any of the following exclusion criteria applied (numbers in parentheses indicate the number of participants who failed each criterion)

- visible fundus abnormality, in the opinion of the optometrist (15)
- abnormal head posture, abnormal ocular motility or ptosis, in the opinion of the orthoptist (19)
- poor visual acuity in either or both eyes with habitual or best correction, defined as acuity worse than 0.2 logMAR on any of the tests performed (84)
- tropia (horizontal or vertical, including intermittent) (11)
- vertical phoria on prism cover test (>0 prism dioptres) (10)
- horizontal phoria on prism cover test of 10 prism dioptres or greater (37)
- interocular difference in visual acuity exceeding 0.2 logMAR with habitual correction (27)
- visual acuity data not recorded (1)

In total, 130/365 participants (36%) were excluded from the “normal” sub-population on this basis. The most common reason for exclusion was poor visual acuity: in 44 out of these 130, poor visual acuity was the only problem detected. Participants tended to show lower acuity with their habitual correction than with their measured best correction. Out of 356 participants for whom both were available, 74/356 had a worst acuity > 0.2 logMAR (worst out of either/both eyes and both distances tested) when tested with their habitual optical correction, compared to only 29/356 when tested with the best correction. We excluded participants from the “normal” population based on their worst acuity score measured with either correction, because the stereo tests were performed with the participants’ habitual optical correction. Thus, people who were capable of good acuity, but who were not wearing the appropriate optical correction to achieve, were placed in our “visual problem” group as well as people whose acuity was poor even with best correction. 8 subjects were excluded solely because of a visual acuity recorded as >0.2logMAR with best correction, even though their visual acuities with habitual correction were all recorded as ≤0.2 (a 9^th^ subject to whom this also applied was excluded already due to a fundus abnormality). We also excluded people who had a large interocular acuity difference when wearing their habitual correction.

The second most common reason for exclusion was horizontal phoria. In 18 out of 130 excluded participants, horizontal phoria in excess of 10 prism dioptres was the only reason for exclusion. A small horizontal phoria was very common, especially at 0.3 m. Out of 253s participants with no other visual problems, 37% had a non-zero horizontal phoria of less than 10 prism dioptres when tested at 0.3 m, whereas only 7% had a horizontal phoria >10. We conclude that a small but measurable horizontal phoria must be considered “normal”, and accordingly only defined as “a problem” horizontal phorias of 10 prism dioptres or greater.

When examining the relationship with stereo thresholds, we also considered a subgroup of specifically binocular vision problems. Participants who met any of the four tropia, phoria, or interocular acuity difference criteria defined above were classed as having a “binocular vision problem” (whether or not they also met any of the other criteria). 68/365 participants had a binocular vision problem by this definition.

### Statistics

As is evident from Figure 2 and Figure 3, the measured stereo thresholds did not conform to the normal distribution. We therefore assessed the significance of differences in median test scores between age/gender groups using a two-tailed Wilcoxon rank-sum test, as implemented in the Matlab function RANKSUM (Matlab R2012a; Mathworks Inc., Sherborn, MA, USA).

To assess whether two groups differed significantly in their proportion of stereonegative participants, we used bootstrap resampling assuming binomial statistics. We first computed the proportion of stereonegatives, s, in both groups pooled. We then used Matlab’s BINORND function to generate sets of n_j_ values with a probability *s* of being stereonegative. In this way, we obtained S_j_ and S_k_ : the proportion of stereonegative results in the resampled data-sets for the two different group-sizes. We compared d = |s_j_–s_k_|, the difference in proportion-stereonegative for the actual age-groups, to D = |S_j_–S_k_|,the difference in the resampled data. We did this for 10,000 sets of resampled data. The proportion of sets on which D exceeded d is the two-tailed significance.

We tested agreement between the stereo classifications on the Frisby and FD2 tests (Table 6) using Cohen’s kappa (without weighting), implemented in Matlab by G. Cardillo (http://www.mathworks.com/matlabcentral/fileexchange/15365).

In Table 7, we used a detection theoretic approach to quantify the association between poor stereovision and other visual problems. In the first four columns, we examined how reliably poor stereovision, defined as a threshold >20arcsec, indicates the presence of the visual problems defined in the Methods. For example, in the first column, “true positives” were participants who had a visual problem and a stereo threshold >20arcsec. “True negatives” were those with no visual problem and a stereo threshold ≤20arcsec. “False positives” had no visual problem, but a stereo threshold >20arcsec, while “false negatives” had a visual problem despite a stereo threshold ≤20 arcsec. True positive rate is then calculated as (number of true positives)/(number of true positives plus false negatives); while true negative rate is (number of true negatives)/(number of true negatives plus false positives). These quantities are analogous to “sensitivity” and “specificity” in the medical literature. In the next two columns, the analysis was similar but only specifically binocular problems were considered (as defined in the previous section). In the final four columns, the analysis is reversed; we examine how reliably visual problems predict poor stereovision. Now, “false positives” were defined as people whose stereo threshold was ≤20arcsec despite a detected visual problem, while “false negatives” were people whose stereo threshold exceeded 20 arcsec despite no detected problem.

**Table 7:** Quantifying how reliably poor stereovision (threshold >20arcsec) is associated with visual problems, either any visual problem or a specifically binocular problem.

	Poor stereovision regarded as indicating visual problems				Visual problems regarded as indicating poor stereovision			
	General problems		Binocular problems		General problems		Binocular problems	
	Frisby	FD2	Frisby	FD2	Frisby	FD2	Frisby	FD2
True positive rate	51%	31%	62%	44%	46%	57%	29%	43%
True negative rate	66%	87%	65%	86%	71%	69%	88%	87%

See Methods (Statistics) for details.

## Results

### Near stereoacuity measured with Frisby test

The distribution of stereo thresholds as measured with the Frisby test is shown in Figure 2, for all participants and for the three age-subgroups. The total height of the bars shows results for the entire population sampled. The black bars show results for the sub-population in which no visual problems were detected. Numbers and medians are reported in Table 1. There were no significant differences between genders in the sample as a whole or in any age-group (Wilcoxon rank-sum test; median = 20 for both males and females; 12 participants whose gender was not recorded were omitted from this analysis).

### Visual factors associated with poor stereoacuity

Table 4 shows the number of participants falling into our three different stereo classes, separated by whether they had a visual problem. Of people with no visual problem, 66% had Class 1 near stereovision, and only 3% had Class 3 stereovision. Of people with a potential visual problem, only 49% had Class 1 stereovision, and 8% had Class 3 stereovision. This is a significant association (p = 0.003sum, χ^2^ test of association).

To quantify the strength of this relationship, we can imagine using Frisby thresholds as a “signal” indicating visual problems. How reliably does poor stereovision indicate underlying visual problems? As Table 7 shows, the data in Table 4 correspond to a true positive rate of 51% and a true negative rate of 66%. That is, only around half of people with a visual problem have poor stereovision, while only two-thirds of people with no detected visual problem have good stereovision. Conversely, we can ask how reliably visual problems are reflected in poor stereovision. The true positive and negative rates are similar (46% and 71%; Table 7). We wondered if this relatively weak relationship was because of the broad definition of “visual problem” adopted in the Methods, some of which would not necessarily be expected to affect binocular vision. For this reason, we also defined a sub-class of specifically binocular problems such as tropia (see Methods for details). However, as Table 7 shows, this did not greatly strengthen the relationship.

We also looked specifically at the participants with the very poorest stereovision. Seven out of 365 participants had a Frisby stereo threshold of 300 arcsec or worse. 6 of these 7 had a tropia (intermittent or otherwise) and the remaining participant had poor visual acuity in one eye (1 logMAR) and no binocular vision. Out of the 4 participants with non-intermittent tropia at 0.3 m, 3 were stereonegative. However, some participants scored well despite tropia. Out of 11 participants with tropia, 5 scored better than 300 arcsec on the Frisby. One participant had a Frisby stereoacuity of 85 arcsec despite having a vertical tropia of 3 prism diopters; another obtained the best available score of 20 arcsec despite having intermittent exotropia and an abnormal “vertical exo” pattern of eye movements. Thus, in our population almost everyone who has a Frisby threshold > 300 arcsec has tropia (true negative rate 99.7%) but half of people with tropia do not score that badly (true positive rate 55%).

### Dependence on age

Previous data have reported that stereoacuity continues to improve up to about age 10, with the most pronounced improvement before age of five [9], [10], [29], [30], [32], [37], and that it declines with later age [12]–[17]. Probably because our data only includes children aged 5 and older, and has a best possible score of 20 arcsec, we do not see a difference between our “visually developing” and “visually mature” groups (Table 1; inset plots in Figure 2), either in stereoacuity or in the proportion of stereonegatives. In the whole population, the “older” group has a higher proportion of stereo-negatives, which is marginally significant (p = 0.046 for “older” vs. “visually developing”, p = 0.03 for “older” vs. “visually mature”, bootstrap resampling), but this difference vanishes if we consider only the sub-population without visual problems. However, the “older” group has significantly worse stereoacuity than either of the other groups, both in the population as a whole and in the sub-population without visual problems (p<0.01 for “older” vs. “visually developing” and for “older” vs “visually mature” in both populations, Wilcoxon rank-sum test on medians). This suggests that while older people can develop specific visual pathologies which abolish stereopsis [16], stereoacuity declines with age independent of other visual problems [15], [34]. There was no evidence in our data of any change in stereoacuity between the ages of 10 and around 50. For example, although in our population as a whole we found a correlation between age and Frisby stereo threshold (Spearman rank correlation *ρ* = 0.1, p = 0.04; N = 365), this was driven by the over-50s. Within our “visually mature” sub-population, there was no correlation between age and Frisby threshold (*ρ* = 0.05, p = 0.36; N = 300).

### Distant stereoacuity measured with FD2 test

Figure 3 shows the distribution of stereo thresholds measured with the FD2 test. Numbers and medians are reported in Table 2.

As for the Frisby stereo test, there were no significant differences between genders. However, there were now proportionally more stereonegative participants in the “visually developing” group (4 out of 35 under-11s tested, all girls aged 7–9) than in the “visually mature” group (p = 0.004 for the whole population, p = 0.04, sub-population with no visual problem; bootstrap resampling). All 4 children who were stereonegative on the FD2 scored 85 arcsec or better on the Frisby, whereas 5 out of 6 over-10s who were stereonegative on the FD2 were also stereonegative on the Frisby, and the 6^th^ had very poor stereo (300 arcsec).

The main reason for this difference seems to be visual problems in children. 3 of the 4 children who were stereonegative on the FD2 had visual problems. Two were from the same family and had poor visual acuity (they were referred for further examination as a result of the study). Previous work has suggested that distance stereoacuity is more impaired by a reduction in visual acuity than near stereoacuity [38], explaining why these children would be selectively impaired on the FD2. The third child had a horizontal phoria of 10 prism diopters at 0.3 m. Even though no phoria was noted at 6 m, it is possible that poor binocular control was responsible for this child’s negative result on the FD2 despite her score of 20 arcsec, the best available, on the Frisby. The fourth child tested negative on FD2 despite the best possible score on the Frisby and no detected visual problems.

A second reason may be that children find the FD2 test harder to understand. Our original data set included 14 children aged from 3 to 7, all of whom were successfully tested on the Frisby test, but 3 of whom (21%) could not be tested with the FD2 because they either did not understand the test or would not cooperate. Thus it is possible that despite apparently demonstrating understanding, some children did not in fact fully understand or attend to the FD2 task, resulting in a false stereonegative.

As with the Frisby, we do not see a significant difference in median FD2 stereo thresholds between the “visually developing” and “visually mature” groups in either the whole population or in the sub-population with no visual problems. However, FD2 stereoacuity in the “older” group is again significantly worse than in either of the other two age-groups (p<0.01 for both pairwise comparisons in the sub-population with no visual problems, and for “visually mature” vs “older” in the whole population; p = 0.03 for “visually developing” vs “older” in the whole population; Wilcoxon rank-sum).

Table 5 shows the association between visual problems and stereo classification on the FD2. As for the Frisby, poor FD2 stereoacuity is weakly associated with visual problems and poor binocular control. Regarded as a signal of binocular problems (see Table 7), the FD2 has a lower rate of true positives (44%) but greater rate of true negatives (87%) than the Frisby.

### Agreement between stereo classification with Frisby and FD2

**Figure 4:**
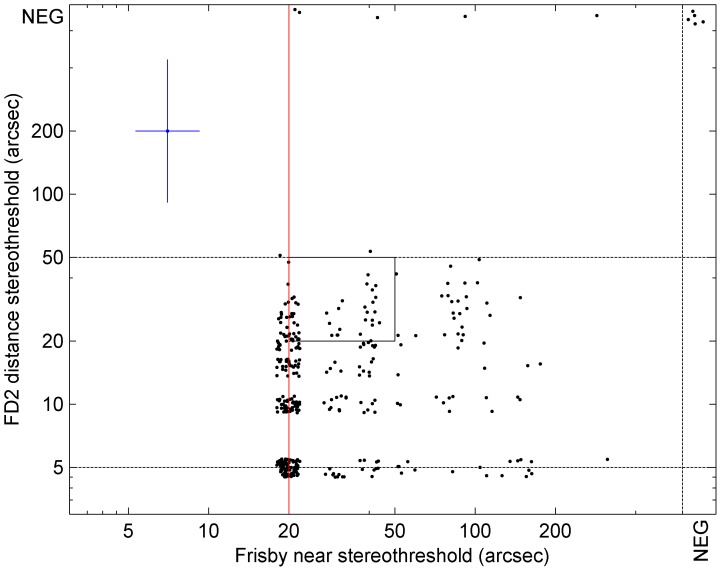
Scatterplot of stereothresholds measured on the revised FD2 against those measured on the Frisby, for all 365 participants. Participants who were stereonegative are plotted at a notional threshold of 700 arcsec. Since possible scores on both tests are quantized, we have jittered the data-points so they do not coincide: for purposes of plotting, each threshold was multiplied by a number between 0.9 and 1.1. The dashed lines indicate the floors and ceilings, i.e. the best and worst thresholds possible on each test. The central rectangle denotes the range of thresholds which were possible scores on both tests. For comparison, the blue cross indicates the 95% limits of agreement reported by Adams et al. [27] for the two tests, i.e. a factor of 1.74 for the near Frisby and 4,8 for the original FD2.

The Frisby and FD2 tests measure different aspects of stereovision. Notably, the very different test distances present different challenges to accommodation and vergence control. Thus, it is not surprising if different stereoacuities are obtained. Additionally the two tests have different ranges. The Frisby test allows scores from 20 arcsec to 600 arcsec (or stereonegative). The FD2 test allows scores from 5 arcsec to 50 arcsec (or stereonegative). Figure 4 shows a scatterplot of results on the two tests for all 365 participants. A Bland-Altman analysis reveals differences between the two tests (Frisby threshold on average 15 arcsec greater than FD2 threshold, *p*<0.001, t-test on differences), but this is largely due to the different ceilings, which are 15 arcsec apart. Viewing Figure 4, it is clear that most participants who scored well on the FD2 also scored well on the Frisby. Four child participants who scored well on the Frisby were classed as stereonegative on the FD2; the possible reasons for this were discussed in the previous section. Overall, the results with the two tests are highly correlated (Spearman correlation r = 0.26, p<0.001). To examine this further, we used each test to classify stereovision into three classes, as described in the Methods. Table 6 compares these classifications for the two tests.

**Figure 5:**
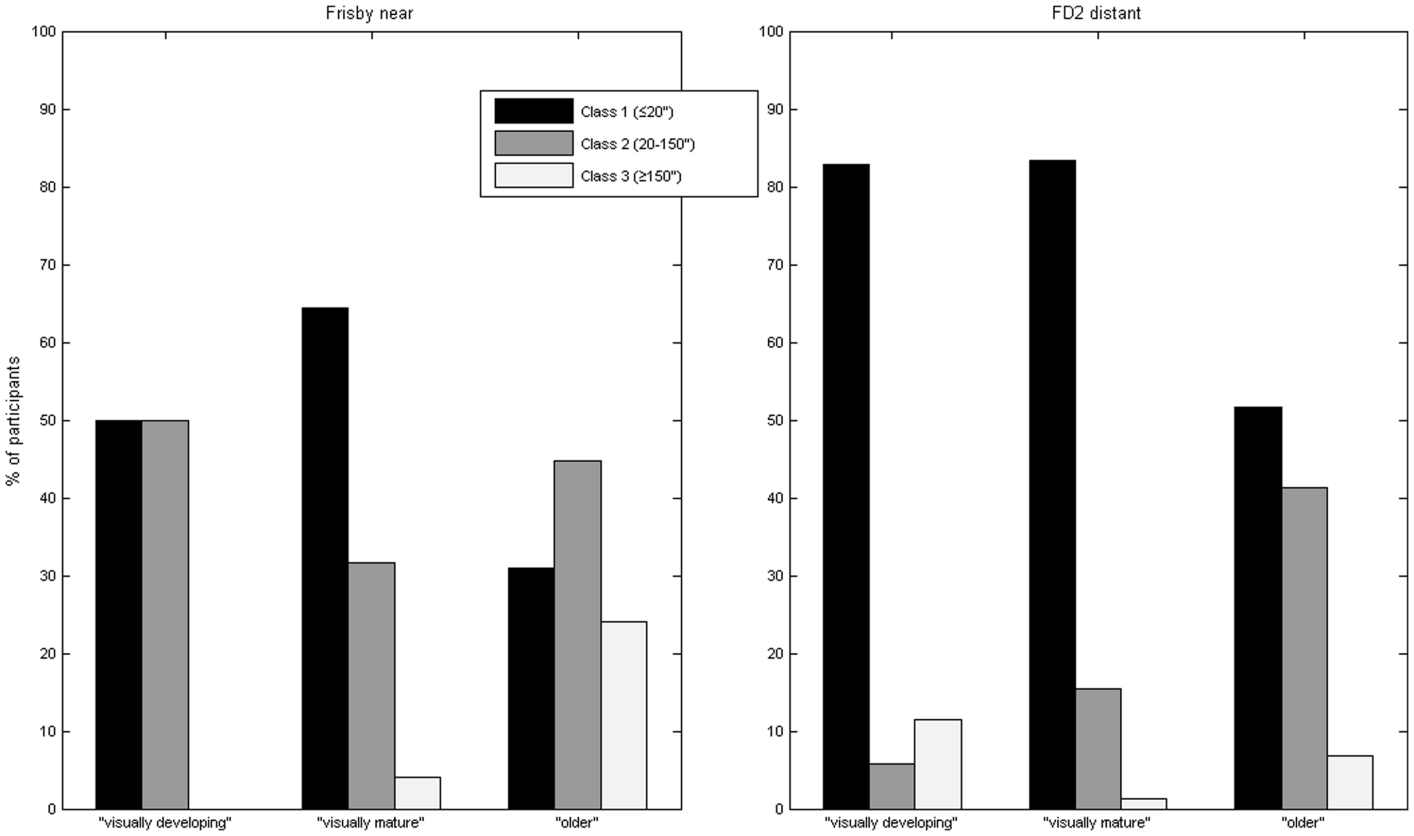
Distribution of our three classes of stereovision in different age groups, as assessed on the two tests.

Figure 5 presents the percentage of participants falling into these 3 classes of stereoacuity in different age-groups. This demonstrates the decline in stereoacuity with age previously remarked upon. For example, no members of the “visually-developing” group were in Class 3 on the Frisby test compared to 24% of “older” participants; 83% of “visually-developing” and “visually-mature” groups had Class 1 stereovision on the FD2, compared to 52% of the “older” group. There was a highly significant association between age and stereo class on both tests (p<0.001, χ^2^-squared test).

Just over half our participants were in Class 1 on both tests (Table 6). The Frisby appears slightly more demanding, in that participants were more likely to fall in Classes 2 or 3 on the Frisby than on the FD2. Out of 293 participants who were in Class 1 under FD2, 98 (33%) were in Classes 2 or 3 on the Frisby. Conversely, out of 219 participants who were in Class 1 on Frisby, only 24 or 11% were in Classes 2 or 3 on the FD2. Cohen’s kappa for classifications on the two tests is 0.24, described as “fair agreement” by Landis & Koch [39]. Thus, despite the major differences between the two tests, they do tend to give fairly similar results when classing the quality of stereopsis.

## Discussion

### Comparison with previously published data

Our range of Frisby and FD2 stereo thresholds are reassuringly similar to those already in the literature. Simons[30] reports a mean Frisby stereaocuity of 251 arcsec for 102 children aged 3–5 years and 143 arcsec for 20 adults (ages unspecified). He used the Frisby test at 40 cm, meaning that there were only 3 possible disparities: 85, 250 and 495 arcsec (note that these are different from the values given in Table 3, presumably because the Frisby test in 1981 used different plate thicknesses). However, he reports that only 8% of child subjects passed the Frisby test’s 85 arcsec plate, although 88% passed the Randot circles test at smaller disparities. We do not have enough participants in this age range for comparison. Even for the adult participants, where our mean Frisby stereoacuity was much lower than reported in Simons[30], the very low ceiling on his data makes it possible that our results are consistent. Simons’ mean score of 143 arcsec could arise from 16/20 participants scoring the best available value of 85 arcsec (the remaining 4 scoring 250 and 495 arcsec in equal numbers). That would mean he finds 75% of adult participants have stereoacuity of 85 arcsec or better, compared to 94% in our dataset (we had 177 adult participants with no visual problems, of whom 167 scored 85 arcsec or less on the Frisby test).

Costa and colleagues[13] studied Frisby stereoacuity in groups aged from 15 to 60 years. Their viewing distance was at least 1 m; they used a track to increase viewing distance in order to obtain arbitrarily small disparities, thus avoiding a ceiling effect. The mean stereoacuity for all age-groups is lower than the 20 arcsec ceiling on our Frisby data. We therefore compare their Frisby results with our FD2 at 6 m. Their mean stereoacuity for 35 subjects aged between 15 and 34 years was 5.8 arcsec (their Table 2; average of crossed and uncrossed disparities and two age-groups). Our 122 visually-normal subjects in this age-range scored worse on the FD2: mean 12.1, median 10 arcsec. However, in the age-range 35–60, Costa et al [13] report a mean stereoacuity of 12.3 arcsec for 11 participants on the Frisby test, very close to our mean of 12.7 arcsec on the FD2 for 60 participants in this age-range and with no visual problems.

Garnham and Sloper[14] measured stereoacuity in 60 visually-normal adult subjects on 4 tests, including the Frisby and FD2 at 6 m. Our results are in very close agreement. Like us, they had a 20 arcsec ceiling on the Frisby. We both find that the median Frisby score in young adults is 20 arcsec, the best score obtainable (median for 31 subjects aged between 17 and 49 in Garnham and Sloper[14]; median for 195 subjects aged between 11 and 49 and with no visual problems in our data-set). We both find that the median FD2 score in this age range is around 10 arcsec (median  =  8 arcsec for Garnham & Slopers’ 31 subjects; median  =  10 arcsec for our 194 subjects). In addition, both studies find a deterioration in the older group (Garnham & Sloper: median threshold  =  40 arcsec on Frisby and 20 on FD2 for 29 subjects aged 50–83; our data-set: median =  75 arcsec on Frisby and 15 on FD2 for 17 subjects aged 50–82).

Leat and co-workers [1] also used the Frisby with a 20 arcsec ceiling. For age-groups 5–7 and 8–20 years, this ceiling was both the mode and the median score (their Table 7). In our data, for 10 children aged 5–7 the mode was 20 and the median was 30 arcsec; for the 83 participants aged 8–20, the mode and median were both 20 arcsec.

Adams and colleagues [28] used the FD2 test on 59 visually-normal children aged between 3 and 5 years. 76% of them were able to perform the FD2 test at 6 m, and these had a mean stereoacuity of 30 arcsec. Our full data-set contains 4 children in this age-range, with stereoacuities of 5, 5, 30 and 35 arcsecs. This gives a mean of 19 arcsec, in good agreement given the small sample size. In accordance with previous studies, our older children (aged 6–10) had better stereoacuity, with a mean of 12 arcsec. This probably reflects cognitive/behavioural factors as well as a genuine improvement in stereoacuity [40].

Hong & Park [32] used the FD2 at 6 m (and 3 m where stereoacuity was poor) to track stereoacuity in visually normal children under 11 years and adults aged 20–39 years. They found that mean stereoacuity was 12.5 arcsec in adults and did not differ significantly in children aged over 5 years. For children, they find slightly better stereoacuity than we do. For 55 children aged 5–10, Hong & Park report a mean FD2 threshold of 14.2 arcsec, whereas we have 36.4 arcsec (22 children aged 5–10 with no visual problems). In the larger adult samples, our results agree closely with theirs. In the age-range 20–39, they report a mean FD2 threshold of 12.5 arcsec (N = 46), while we have 12.1 arcsec (114 adults aged 20–39 with no visual problems).

In summary, our results are in close agreement with previous studies on both the Frisby and FD2 tests. This indicates that values obtained with the modified FD2 do not differ significantly from those obtained with the original version of the test.

### Dependence on age

In line with the literature, we observed a decline in stereopsis for older adults, not explained solely by visual abnormalities (Figure 2BCD; Figure 3C vs. D; Figure 5). We did not observe significant differences between the “visually developing” and “visually mature” groups, probably because our “developing” group was relatively small and began after the most rapid period of improvement in stereovision. Children seemed to find the FD2 test cognitively more demanding than the Frisby, possibly because it is more difficult to direct a small child’s attention to something several meters away than to something right in front of them. No children failed to demonstrate understanding of the Frisby, but a few did on the FD2. Children were also more likely to be stereonegative on the FD2

### Stereoblindness

The term stereoblindness is generally used to mean the complete absence of stereopsis. Reported figures for stereoblindness vary widely, with estimates as high as 30% in some sources. A recent report from the 1958 British birth cohort [41], studying 9330 people aged 44–45 years, found that 14% had stereo thresholds > 400 arcsec using the Lang II stereocard; even after excluding those known to have had previous treatment for strabismus, the figure was still 12%. In our study, including a wide age-range and people with known visual problems, fewer than 2% of participants did not achieve a threshold of 150 arcsec on either test.

Previous work has shown that “stereoblindness” depends strongly on the particular test employed. In general, participants score better on “real depth” tests such as those used here than on “pure disparity” tests such as random-dot patterns, and our results are consistent with this [38]. Conversely, distance stereoacuity in prism-induced convergence stress degrades more rapidly for the “real depth” FD2 than the “pure disparity” Distance Randot [42], [43]. The effects of age may also be less pronounced on “real depth” tasks [14]. The reasons for these differences are still unclear. An obvious concern would be that the “real depth” tests allow participants to use non-stereo cues, and yet controls with monocular viewing suggest that this is not the reason for the discrepancy.

### Summary

In line with previous literature, our results suggest that stereovision improves up to the age of around 10 or 11 and then shows little change until age 50 or so. Thereafter, an age-related decline in stereoacuity occurs, apparently not simply as a side effect of other visual problems of ageing.

Poor stereovision was weakly associated with binocular problems such as tropia. In our population, around 1 in 5 people was identified as having some form of binocular problem. Of people with Frisby thresholds > 20 arcsec, this rose to 2 in 3. However, around 1 in 3 people with a binocular problem still scored the best available Frisby threshold of 20 arcsec. Of people with FD2 thresholds >20arcsec, around half had a binocular problem, and only 1 in 8 people with a binocular problem scored better than 20 arcsec on the FD2. This confirms that binocular problems are a common reason for poor stereovision, but around a third of people with poor stereovision don’t appear to have any such reason for it. It is unusual for people with binocular problem to have good stereovision, especially on the more demanding FD2 test, but certainly possible.

Finally, the revised version of the FD2 test, without a backlight, appears to give very similar results to the original version. We therefore conclude the revised version can be used without hesitation.
